# Case Report: Persistent residual shunt after a first percutaneous PFO closure followed by minimally invasive surgical failure: third time is a charm

**DOI:** 10.3389/fcvm.2024.1367515

**Published:** 2024-07-02

**Authors:** Eustaquio M. Onorato, Francesco Alamanni, Giovanni Monizzi, Angelo Mastrangelo, Antonio Luca Bartorelli

**Affiliations:** ^1^ University Cardiology Department, I.R.C.C.S. Ospedale Galeazzi- Sant’Ambrogio, Milan, Italy; ^2^ University Cardiac Surgery Department, I.R.C.C.S. Ospedale Galeazzi- Sant’Ambrogio, Milan, Italy; ^3^ Department of Biomedical and Clinical Sciences, “Luigi Sacco”, University of Milan, Milan, Italy

**Keywords:** patent foramen ovale, transcatheter closure, atrial septal aneurysm, multifenestrated atrial septum aneurysm, residual shunt, minimally invasive cardiac surgery

## Abstract

**Background:**

Even though the optimal management of a moderate or large residual shunt following patent foramen ovale (PFO) closure is open to question, recent data confirmed that it is associated with an increased risk of stroke recurrence.

**Case summary:**

A 48-year-old woman, a migraineur with visual aura, was diagnosed with a PFO associated with a huge multifenestrated atrial septal aneurysm (mfASA) and a moderate right-to-left shunt, detectable only after a Valsalva maneuver on contrast-transthoracic echocardiography. Brain magnetic resonance imaging showed a 1-mm silent white matter lesion in the right frontal lobe. Although the indication was not supported by guidelines, a transcatheter PFO closure was performed at another center with implantation of a large, equally sized, double-disc device (Figulla UNI 33/33 mm). At 6-month follow-up, a 2D/3D transesophageal echocardiography (TEE) color Doppler showed incorrect orientation of the device, which was not parallel to the interatrial septum, with two discs failing to capture the aortic muscular rim and partially protruding in the right atrium; furthermore, a 4 mm × 7 mm ASA fenestration was documented with a residual bidirectional shunt. Thereafter, the same team performed a minimally invasive cardiac surgery under femoro-femoral cardiopulmonary bypass; however, the procedure proved ineffective and was complicated by postoperative pericarditis with pericardial effusion, requiring further rehospitalization 1 month later due to persistent pericarditis, bilateral pleuritis, phrenic nerve palsy, and atrial flutter, which was treated with amiodarone. The patient asked for a second opinion, and our multidisciplinary heart team decided to offer a percutaneous redo intervention. An uneventful implantation of a regular PFO occluder (Figulla Flex II 16/18 mm) across the septal defect was performed successfully. Twelve-month follow-up with 2D TTE color Doppler and contrast transcranial Doppler showed correct position and good interaction between the two devices, with no residual shunt.

**Discussion:**

In addition to the incorrect indication for PFO closure and the failure of minimally invasive surgery, the procedural mishap in this case could have been due to the inappropriate implantation of the first large device within the tunnel. It would have been better to deploy the same large device in the most central fenestration, covering the PFO and a greater part of the remaining mfASA at the same time.

## Introduction

Transcatheter patent foramen ovale (PFO) closure is a safe, effective, and highly successful procedure, associated with a low incidence of in-hospital complications and a low frequency of recurrent ischemic events at long-term follow-up; the risk of recurrence increases with the grade of the residual shunt (1–3). Complications related to the procedure and the device have been very low and generally transient (4). Furthermore, the selection of appropriate patients and devices suitable for the corresponding anatomical features of the PFO is essential for effective closure (5).

Moderate-to-severe residual right-to-left shunt (RLS) after PFO closure has been reported in approximately 10% of patients (6, 7) and has been associated with an increased risk of recurrent stroke or transient ischemic attack in long-term follow-up.

Until now, no agreement has been reached regarding the best management of a persisting residual shunt. Despite the lack of long-term data, a second transcatheter procedure appears to be technically feasible and safe, avoiding a more invasive surgical procedure. Several reports in the literature describe the implantation of a second device achieving complete closure (8–10).

We describe a case of persistent residual shunt after percutaneous PFO closure, followed by a minimal invasive surgical failure in a patient for whom a third procedure using a second device was successful, resulting in good clinical outcomes thereafter.

## Case presentation

We present a case of a 48-year-old woman, a nurse and sportswoman, in whom contrast 2D transesophageal echocardiography (TTE) color Doppler demonstrated the presence of a tunnel-like PFO associated with huge mfASA and moderate RLS, visible only after the Valsalva maneuver. Brain magnetic resonance imaging (MRI) showed a 1-mm, silent, non-specific white matter lesion in the right frontal lobe. Her past medical history was unremarkable, except for episodic migraines with a visual aura that were responsive to ibuprofen. No thrombophilic disorders and atrial fibrillation were reported.

**Figure 1:**
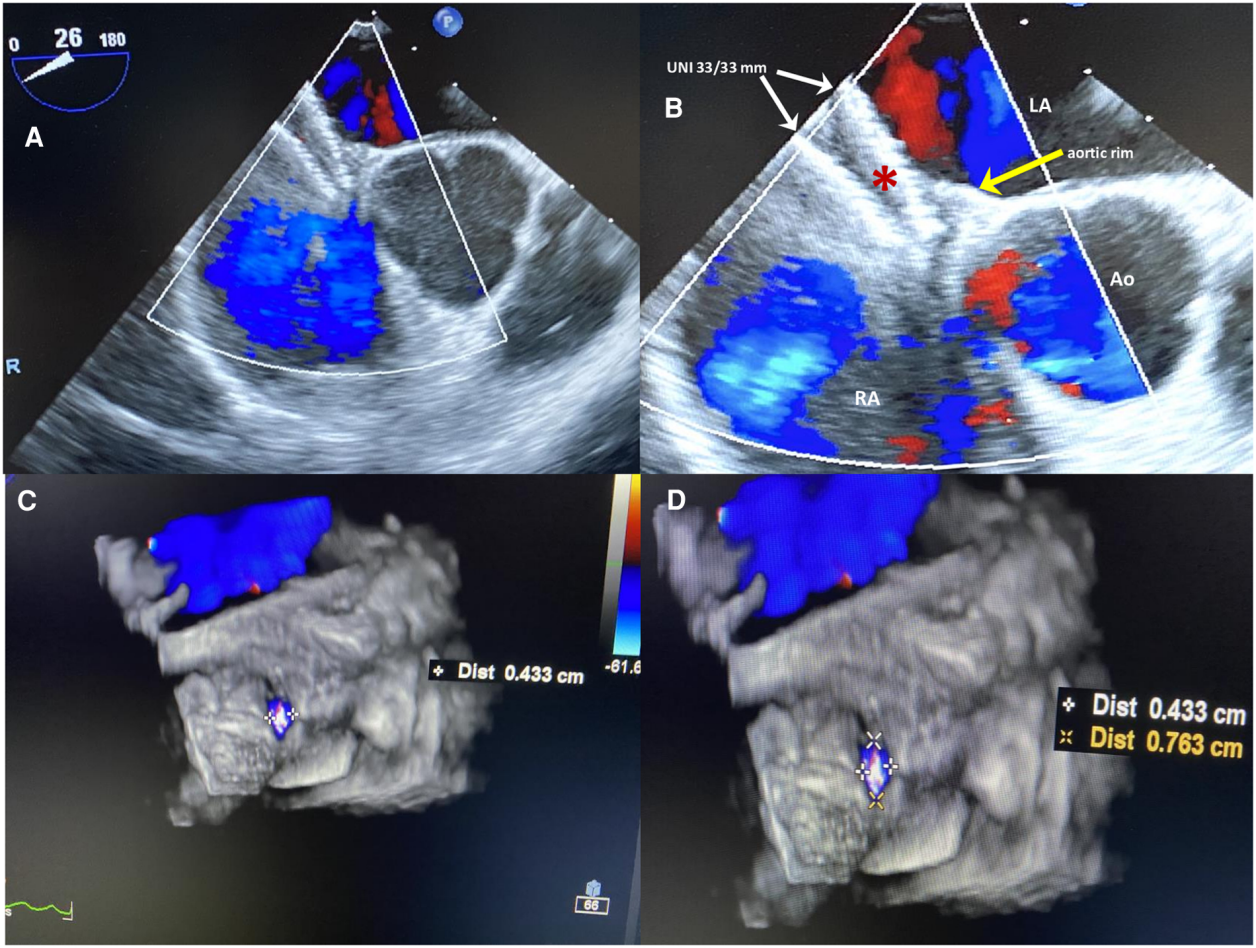
(**A,B**) Six-months 2D TEE color Doppler in the short view after the first percutaneous PFO closure procedure. The large equally sized double-disc UNI 33/33 mm device (red asterisk), is not parallel to the interatrial septum, with two discs diverging on one side (white arrows) and on the other side not capturing the aortic muscular rim (yellow arrow); the device is partially protruding in the right atrium with inappropriate orientation. (**C,D**) 3D TEE color Doppler showing a 4 mm × 7 mm ASA fenestration (septal defect) far from the UNI device.

Although the indication was not supported by the guidelines (11, 12), transcatheter PFO closure was performed at another center with the implantation of a large, equally sized, double-disc device, Figulla UNI 33/33 mm (Occlutech International AB, Helsinborg, Sweden). The patient was discharged home on dual antiplatelet therapy (aspirin 100 mg and clopidogrel 75 mg daily) for 6 months. At 6 months follow-up, 2D/3D transesophageal echocardiography (TEE) color Doppler showed incorrect orientation of the device, not parallel to the interatrial septum, with the two discs not capturing the aortic muscular rim and partially protruding in the right atrium (Figures 1A,B); furthermore, a 4 mm × 7 mm ASA fenestration (septal defect) far from the UNI device was also identified (Figures 1C,D), with a residual bidirectional shunt that persisted unaltered on subsequent controls. Eight months later, the same team performed a minimally invasive cardiac surgery (MICS) procedure on the patient using a right parasternal approach under femoro-femoral cardiopulmonary bypass, which failed to close the residual shunt with interrupted sutures, ultimately leaving the inappropriately oriented device *in situ*. Regrettably, surgery proved not only ineffective but also complicated by postoperative pericarditis with pericardial effusion, which prolonged hospital stay. One month later, the patient was re-hospitalized due to persistent pericarditis, bilateral pleuritis, phrenic nerve palsy, and atrial flutter and was treated with amiodarone. Thereafter, colchicine and high doses of prednisone were used to treat and prevent recurrent pericarditis.

The patient continued to complain of malaise, asthenia, and palpitations. Therefore, ergometric testing and stress echocardiography were performed, which showed normal results; no arrhythmias were recorded on the ECG Holter. Multidetector computed tomography angiography ruled out pulmonary thromboembolism. Moderate lung function impairment was detected, characterized by a reduction in forced expiratory volume (FEV1) and forced vital capacity (FVC). Meantime, the patient was unable to resume her nursing responsibilities and sporting activities due to persistent dyspnea on exertion and tiredness. She finally decided to attend our Heart and Brain Clinic a year later for a second opinion; after evaluation, our multidisciplinary heart team decided to offer a percutaneous redo intervention due to deteriorating clinical conditions and the persistent significant residual shunt, which was confirmed by contrast 2D TTE (Supplementary Figure S1). Written informed consent for a redo procedure was obtained from the patient. During her third hospitalization, the electrocardiogram (ECG) showed an incomplete right bundle branch block, and continuous ECG monitoring ruled out atrial fibrillation. A chest x-ray revealed no signs of increased pulmonary flow. The procedure was carried out under local anesthesia, fluoroscopic guidance, and continuous rotational intracardiac echocardiography by an Ultra ICE (EP Technologies, Boston Scientific Corporation, San Jose, CA, USA), as previously described (13), using two standardized sections: a transverse one on the aortic valve plane and a longitudinal section on the four-chamber plane (14).

**Figure 2:**
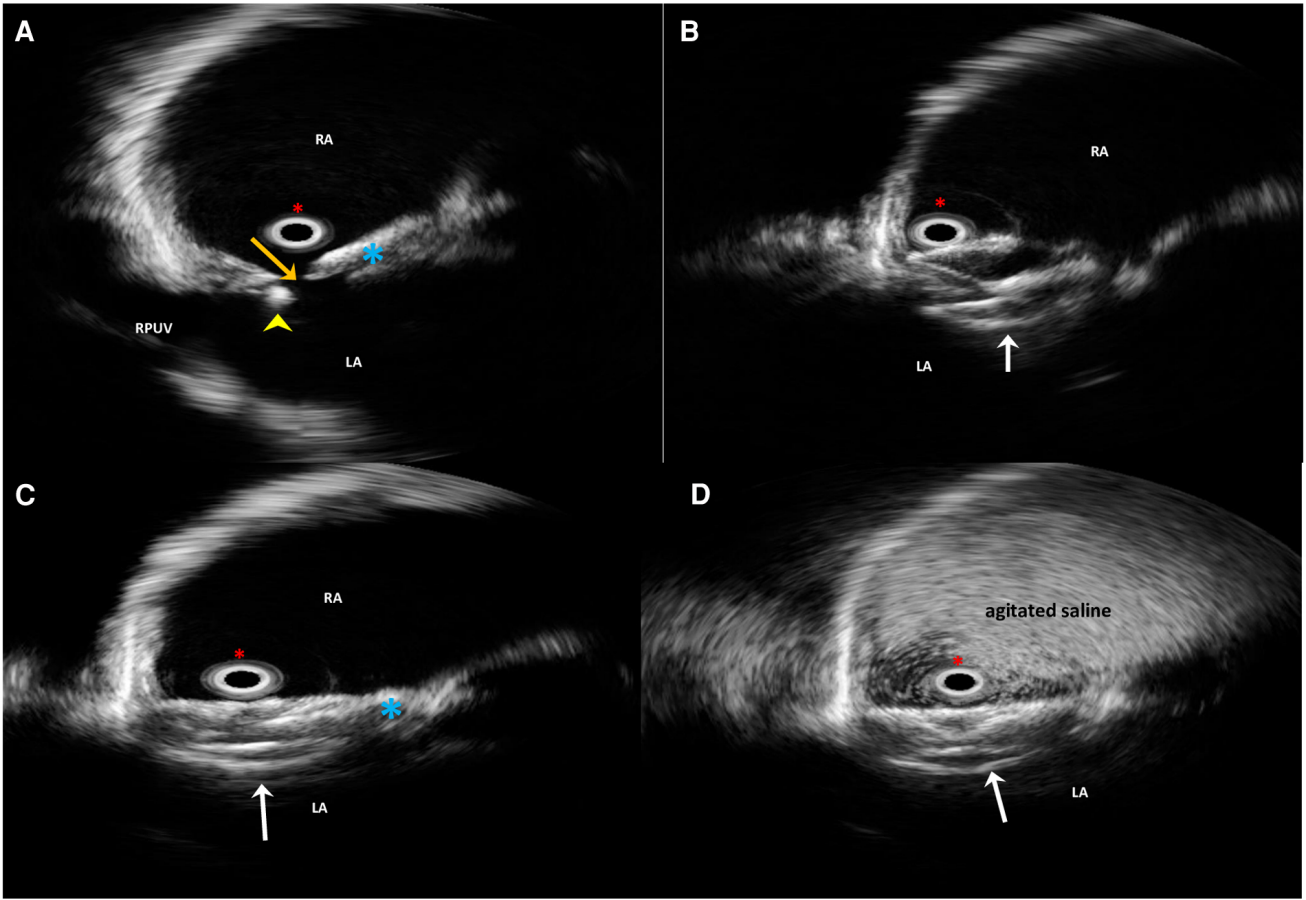
Long-axis four-chamber plane intraprocedural rotational intracardiac echocardiography by Ultra ICE (mechanical 9F/9 MHz 360° scan probe) procedural steps. The guidewire (yellow arrowhead) is across the residual septal defect (orange arrow) apart from the previously implanted device (light blue asterisk) (**A**). Successful implantation steps of the Flex II PFO 16/18-mm device (white arrows) (**B**,**C**). Contrast-enhanced Ultra ICE image confirming the abolition of the residual shunt (**D**). LA, left atrium; RA, right atrium; RUPV, right upper pulmonary vein. Red asterisk, the Ultra ICE-9F-9 MHz catheter located at the center of the image.

**Figure 3:**
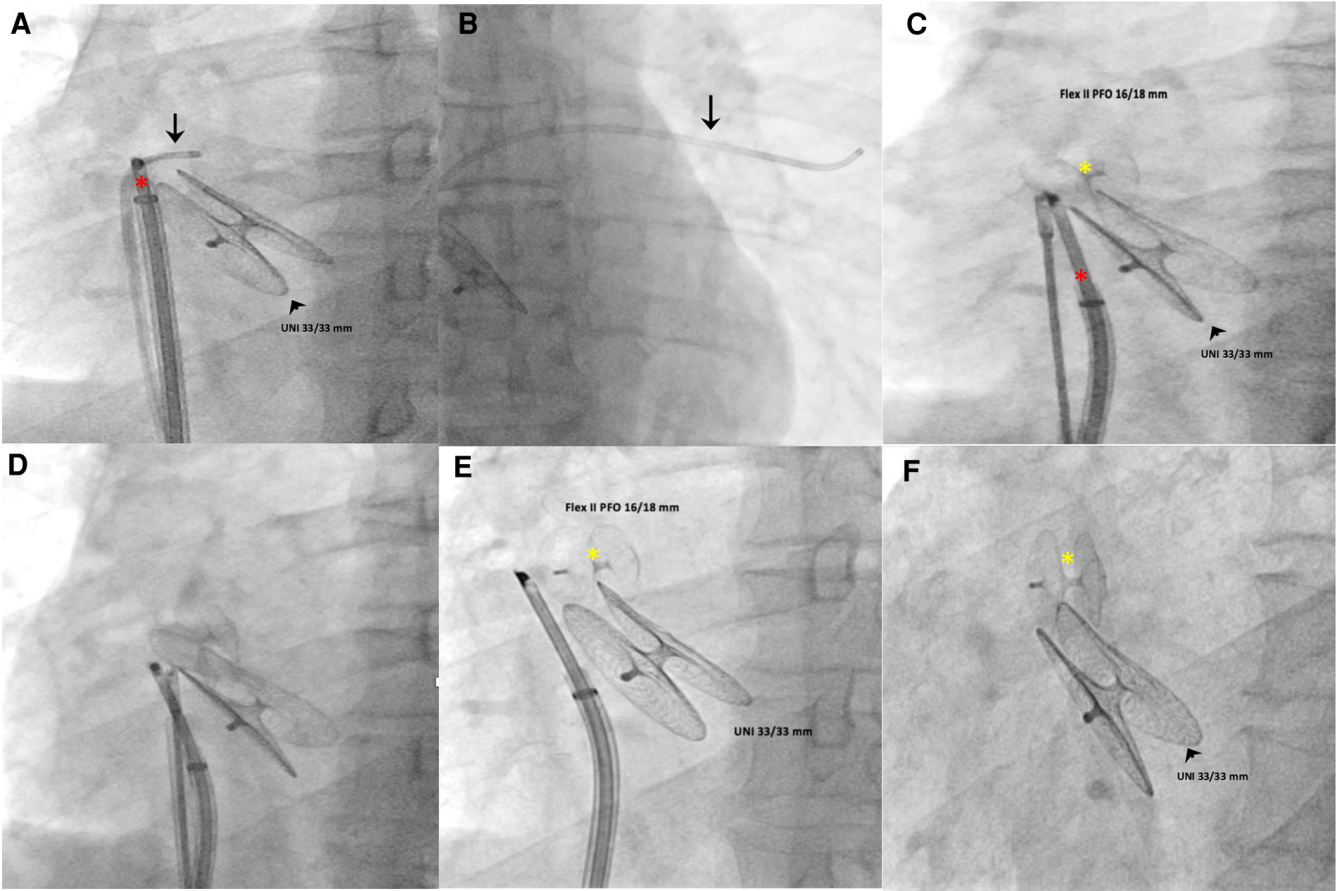
Intraprocedural fluoroscopic procedural steps (left anterior oblique 30° view). A multipurpose catheter (black arrow) crossed the septal fenestration and was then positioned in the upper left pulmonary vein (**A**,**B**). Using a dedicated 9-Fr-long sheath, a PFO occluder (Figulla Flex II 16/18 mm) was advanced across the septal fenestration and deployed in the appropriate orientation without impinging the previously implanted device (**C**–**F**). The red asterisk indicates the Ultra ICE-9F-9 MHz catheter; black arrowhead, UNI 33/33 mm; yellow asterisk, Figulla Flex II 16/18 mm.

**Figure 4:**
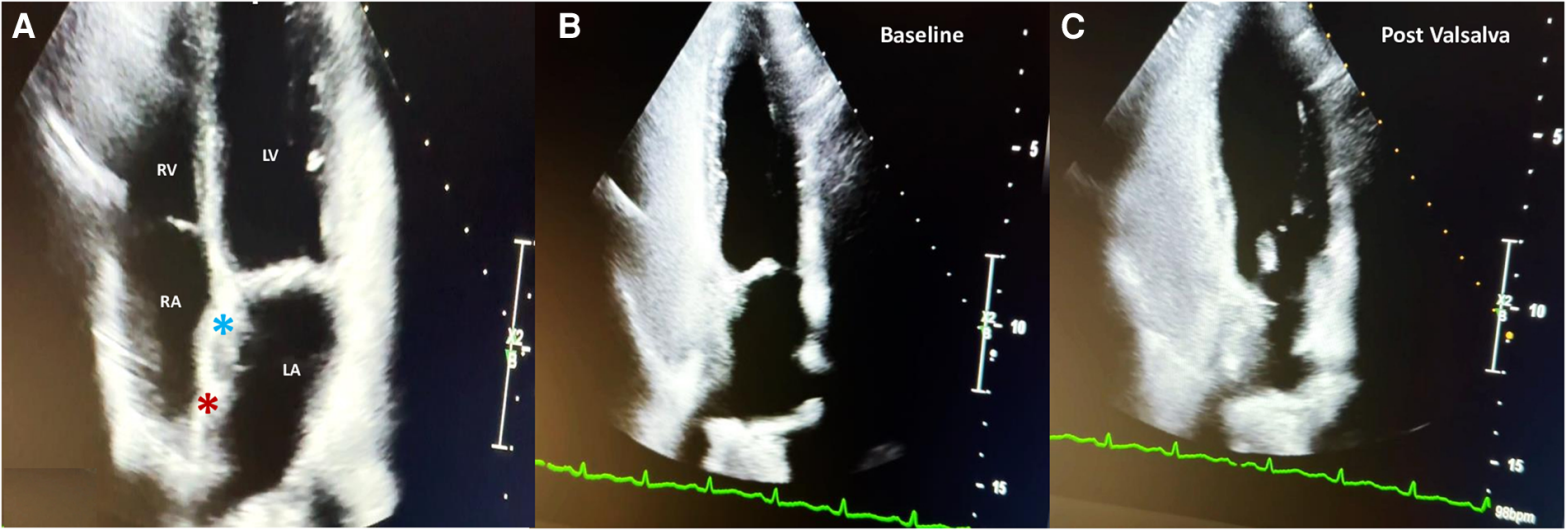
Two-year follow-up 2D TTE color Doppler in the apical four-chamber view showing the correct interaction between the UNI 33/33-mm device (light blue asterisk) and the Figulla Flex II 16/18-mm device (red asterisk) (**A**); contrast 2D TTE confirming abolition of the residual shunt at baseline (**B**) and after the Valsalva maneuver (**C**).

Access to the right and left femoral veins was obtained using 8-Fr short introducer sheaths. Thereafter, the septal fenestration, apart from the previously implanted device, was crossed by a 6-Fr multipurpose catheter. After successful placement of a 260-cm exchange guidewire in the upper left pulmonary vein, the dedicated 9-Fr delivery sheath was advanced over the wire into the left atrium. The distal disc of the PFO Figulla Flex II 16/18 mm (Occlutech International AB, Sweden) was then opened in the left atrium and pulled back against the septum at the edge of the previously implanted UNI 33/33 mm device. While maintaining tension on the delivery cable, the proximal disc was then opened in the right atrium and pushed forward to the septal fenestration. After a meticulous “push-and-pull” maneuver, the device was successfully released, and fluoroscopic and rotational intracardiac ultrasound guidance imaging showed the two devices in profile without interferences or malapposition/dislocations between them (Figures 2,3). The patient was discharged home the following day in good clinical condition. Dual antiplatelet therapy (aspirin 100 mg/daily and clopidogrel 75 mg/daily) was recommended for the first 2 months, followed by single antiplatelet therapy (aspirin 100 mg/daily) up to 6 months. Antibiotic prophylaxis for infective endocarditis was also suggested. Follow-up with 2D TTE color Doppler and contrast transcranial Doppler (cTCD) at 6 and 12 months confirmed the correct position and good interaction between the two devices, without any residual shunt (Figure 4, Supplementary Figure S2). Currently, clinical improvement has been achieved, providing a better quality of life.

## Discussion

Among the different types of residual shunts after PFO closure, the most common is the in-tunnel shunt between the devices’ discs. Another type of residual shunt may be due to iatrogenic erosion at the edge of device rims, resulting in a small atrial septal defect that can be responsible for left-to-right or bidirectional shunts, with or without hemodynamic impairment (15, 16).

Furthermore, accessory undetected multiple fenestrations of ASA associated with PFO, as in our case, are another frequent but avoidable cause of a significant residual shunt after PFO closure; their presence should be ruled out at during baseline echocardiographic evaluation or at the time of the procedure (17).

Some anatomical conditions are proven independent predictors of residual shunt after percutaneous PFO closure (18–21). Among them, ASA, defined as an excursion ≥10 mm with a base diameter ≥15 mm of the septum primum, is considered one of the key features of complex PFO anatomies. In the majority of cases, it is frequently associated with moderate-to-severe baseline RLS and a fourfold higher risk of paradoxical events (22).

Implantation of larger devices to cover the vast majority of the aneurysm is a controversial strategy still adopted in many centers, resulting in additional risk factor for the occurrence of a residual shunt (9). Indeed, a bigger device does not always guarantee the effective closure of the multifenestrations, and the choice of implanting a single larger device through the largest fenestration or, alternatively, two smaller occluders instead of a single larger one may prove successful for this purpose.

In fact, the presence of a residual shunt after PFO closure has been a topic of ongoing debate for many years, primarily due to the controversy regarding its association with an increased risk of recurrent ischemic events (23–25).

Recently, a prospective cohort study aimed at comparing the recurrence of ischemic cerebral events in those with and without a residual shunt has been published, confirming that residual shunt was associated with a significant increased risk of recurrent event and the risk was heightened in patients with higher grades of the residual shunt (26). Moreover, the 2021 American Guidelines for the Prevention of Stroke reached the same conclusion, confirming that residual shunt is associated with an increased risk of stroke recurrence (27).

Even though the optimal management of residual moderate or large residual shunt after PFO closure remains open to question (20, 28), the literature on long-term results after the implantation of a second device is increasing, and retrospective evaluations are encouraging (8–10, 29, 30).

The paradigm of the modern era of cardiac surgery is changing with the use of MICS, which allows performing a wide variety of complex operations (valve repair, valve replacement, and coronary artery bypass graft surgery) through right or left mini-thoracotomy (sternotomy-free) and, in some cases, without cardiopulmonary bypass (31–34). Nevertheless, the risks associated with minimally invasive heart surgery are similar to those of open-heart surgery, including bleeding, infection, stroke, pericarditis, pleuritis, and phrenic nerve traction injury. Furthermore, these procedures may be more expensive and take longer to perform.

Although performed entirely through a mini-thoracotomy in the right intercostal space, minimizing surgical trauma, allowing quick recovery, and offering excellent cosmetic results, MICS may not be the best choice for treating residual shunts. In fact, in our patient, it was not only ineffective but also complicated by pericarditis, pleuritis, and phrenic nerve palsy. Therefore, a second percutaneous closure procedure, less invasive than surgery, should be strongly considered for patients with moderate-to-large residual shunts and appears to be technically feasible, effective, and safe. Moreover, to improve procedural success, a better understanding of the anatomic and device-related factors associated with closure efficacy is needed (17, 18).

In addition to the incorrect PFO closure indication and the ineffective and risky minimally invasive surgery, procedural failure in this case could have been due to the inappropriate implantation of the first large device within the PFO tunnel. Given the complex morphology of the PFO associated with mfASA, it would have been better to deploy the same large device in the most central fenestration, covering the PFO and a great part of the remaining mfASA at the same time.

## Conclusion

Moderate-to-large residual shunts after PFO closure represent technical- or procedure-related failures that may be due to complex, unsuitable PFO anatomies and inaccurate selection of devices in the majority of cases. This complication needs further treatment, percutaneously or surgically, due to the risk of recurrent embolic events. The present case confirms that a second transcatheter closure of a residual shunt should be the first-line treatment option because it is technically feasible, effective, safe, and less invasive than surgery.

## Data Availability

The raw data supporting the conclusions of this article will be made available by the authors without undue reservation.

## Supplementary material

Supplementary Figure S1 Contrast 2D TTE in the apical four-chamber view showing a moderate right-to-left shunt at baseline (**A**) that increases significantly after the Valsalva maneuver (**B**).

Supplementary Figure S2 Two-year follow-up contrast-enhanced transcranial Doppler at baseline (**A**) and after the decisive Valsalva maneuver (**B**) showing no RLS at all.
