# Supplementary material for: Case Report: Persistent residual shunt after a first percutaneous PFO closure followed by minimally invasive surgical failure: third time is a charm
Source: Front Cardiovasc Med. 2024 Jul 2;11:1367515. doi: 10.3389/fcvm.2024.1367515 (PMC11249728; doi:10.3389/fcvm.2024.1367515)
Supplement: Supplementary file 1 [file Datasheet1.pdf]

TIMELINE

Date	Event
2005	Episodic migraine with visual aura responsive to ibuprofen
January 2018	Contrast 2D TTE color Doppler demonstrated the presence of a tunnel-like PFO associated with huge mfASA with moderate RLS only after Valsalva maneuver
March 2018	Brain MRI showed one-millimeter silent aspecific white matter lesion of the right frontal lobe
June 2019	Transcatheter PFO closure was performed with implantation of a large, equally sized, double-disc device Figulla UNI 33/33 mm
December 2019	2D/3D TEE color Doppler showed incorrect orientation of the device, not parallel to the interatrial septum, with two discs not capturing the aortic muscular rim; furthermore, a 4x7 mm ASA fenestration (septal defect) far from the UNI device was also shown, with residual bidirectional shunt
February 2020	Minimally invasive cardiac surgery failure under femoro-femoral cardiopulmonary bypass using right parasternal approach complicated by postoperative pericardial effusion
April 2020	Re-hospitalization due to persisting pericarditis, bilateral pleuritis, phrenic nerve palsy and atrial flutter<br><br>Colchicine associated with high doses of prednisone were used to treat and prevent recurrent pericarditis
October 2021	An uneventful implantation of a regular PFO occluder (Figulla Flex II 16/18 mm) across the ASA fenestration was performed
November 2022	2D TTE color Doppler and cTCD follow-up confirmed correct position and good interaction between the two devices in the absence of any residual shunt<br><br>Better clinical conditions and improved quality of life
