# Supplementary figures and images for: Case Report: Persistent residual shunt after a first percutaneous PFO closure followed by minimally invasive surgical failure: third time is a charm
Source: Front Cardiovasc Med. 2024 Jul 2;11:1367515. doi: 10.3389/fcvm.2024.1367515 (PMC11249728; doi:10.3389/fcvm.2024.1367515)

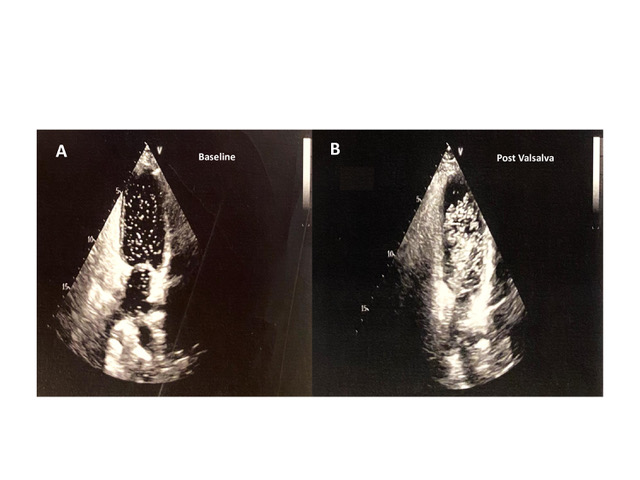

Supplement: Supplementary Figure S1 — Contrast 2D TTE in the apical four-chamber view showing a moderate right-to-left shunt at baseline (A) that increases significantly after the Valsalva maneuver (B). [file Image1.jpeg]

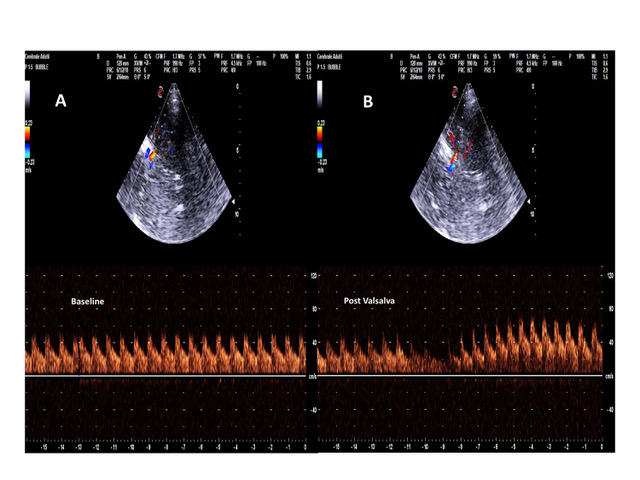

Supplement: Supplementary Figure S2 — Two-year follow-up contrast-enhanced transcranial Doppler at baseline (A) and after the decisive Valsalva maneuver (B) showing no RLS at all. [file Image2.jpeg]
